# Accuracy and Reliability of a Suite of Digital Measures of Walking Generated Using a Wrist-Worn Sensor in Healthy Individuals: Performance Characterization Study

**DOI:** 10.2196/48270

**Published:** 2023-08-03

**Authors:** Nathan Kowahl, Sooyoon Shin, Poulami Barman, Erin Rainaldi, Sara Popham, Ritu Kapur

**Affiliations:** 1 Verily Life Sciences South San Francisco, CA United States

**Keywords:** digital measurements, wearable technology, mobility measurements, walking patterns, wearable, wearables, sensor, sensors, mobility, measurement, measurements, walk, walking, gait, step, wrist-worn, reliability, accuracy

## Abstract

**Background:**

Mobility is a meaningful aspect of an individual’s health whose quantification can provide clinical insights. Wearable sensor technology can quantify walking behaviors (a key aspect of mobility) through continuous passive monitoring.

**Objective:**

Our objective was to characterize the analytical performance (accuracy and reliability) of a suite of digital measures of walking behaviors as critical aspects in the practical implementation of digital measures into clinical studies.

**Methods:**

We collected data from a wrist-worn device (the Verily Study Watch) worn for multiple days by a cohort of volunteer participants without a history of gait or walking impairment in a real-world setting. On the basis of step measurements computed in 10-second epochs from sensor data, we generated individual daily aggregates (participant-days) to derive a suite of measures of walking: step count, walking bout duration, number of total walking bouts, number of long walking bouts, number of short walking bouts, peak 30-minute walking cadence, and peak 30-minute walking pace. To characterize the accuracy of the measures, we examined agreement with truth labels generated by a concurrent, ankle-worn, reference device (Modus StepWatch 4) with known low error, calculating the following metrics: intraclass correlation coefficient (ICC), Pearson *r* coefficient, mean error, and mean absolute error. To characterize the reliability, we developed a novel approach to identify the time to reach a reliable readout (time to reliability) for each measure. This was accomplished by computing mean values over aggregation scopes ranging from 1 to 30 days and analyzing test-retest reliability based on ICCs between adjacent (nonoverlapping) time windows for each measure.

**Results:**

In the accuracy characterization, we collected data for a total of 162 participant-days from a testing cohort (n=35 participants; median observation time 5 days). Agreement with the reference device–based readouts in the testing subcohort (n=35) for the 8 measurements under evaluation, as reflected by ICCs, ranged between 0.7 and 0.9; Pearson *r* values were all greater than 0.75, and all reached statistical significance (*P*<.001). For the time-to-reliability characterization, we collected data for a total of 15,120 participant-days (overall cohort N=234; median observation time 119 days). All digital measures achieved an ICC between adjacent readouts of >0.75 by 16 days of wear time.

**Conclusions:**

We characterized the accuracy and reliability of a suite of digital measures that provides comprehensive information about walking behaviors in real-world settings. These results, which report the level of agreement with high-accuracy reference labels and the time duration required to establish reliable measure readouts, can guide the practical implementation of these measures into clinical studies. Well-characterized tools to quantify walking behaviors in research contexts can provide valuable clinical information about general population cohorts and patients with specific conditions.

## Introduction

Assessing an individual’s mobility can provide meaningful insights into their general health status. In clinical settings, mobility is a fundamental factor to define prognosis and care as it is closely associated with a wide array of health outcomes [1-3]. However, accurate and reliable quantification of mobility in real-world settings remains challenging because self-reported data from instruments such as the International Physical Activity Questionnaire can be biased by limited recall and social desirability [4,5].

The interest in quantifying physical activity using wearable devices has recently increased, as these technologies can collect objective individualized data [6]. Wearable sensors have been incorporated into clinical studies across different disease states to enable movement analyses and the quantification of discrete physical activities to develop clinically meaningful end points [7,8].

Yet, to cement their research utility, two aspects of these digital measurements need to be properly characterized: (1) the accuracy with which a digital measurement reads the parameters of interest [9] and (2) the amount of aggregated data needed to reliably capture an individual’s underlying behavioral state, minimizing noise related to natural variability, which usually translates into an aggregation time period for data collection (time to reliability). Although accuracy is always a critical aspect in the characterization of a measure’s performance, time to reliability tends to be ignored, even though it is key for establishing fundamental study design specifications (eg, collection time periods and length of wear time per day), defining baselines, or computing power calculations for the detection of intervention effects or other changes.

Studies characterizing the performance of digital walking measures often focus on step count. However, the literature around these studies shows considerable heterogeneity across designs and some notable limitations. First, analyses tend to rely on truth labels originated by participants’ self-reports, short-term close monitoring [10-12], or from reference devices with suboptimal accuracy (mean absolute percentage error >20%) and with the same body placement as the investigational devices, which would bias agreement results [13]. Second, these studies are often conducted in artificial laboratory environments, which inherently limit behavior range and are susceptible to subjectivity, assessment bias, and unreliability [14-16]. Third, reliability characterization for investigational digital measurements is often absent from studies, despite having been acknowledged as an important element for the validation of clinically important research metrics, such as patient-reported outcomes [17]. Beyond step counts, there have been studies that have used other digital measures (eg, walking intensity captured by the peak 30-minute cadence) to generate clinical insights but without full characterization of their performance [18-29].

In a previous study, we developed an algorithm that accurately classifies ambulatory status from data collected from a wrist-worn device, characterizing its performance across diverse demographic groups in a real-world setting [30]. Further, results from a substudy of an interventional randomized phase 2 trial demonstrated that digital measures of physical activity (step count and ambulatory time) could be sensitive to treatment effects in patients with Lewy body dementia [31].

Herein, we report on the development of a series of measures that can capture walking behavior comprehensively, characterizing their analytical performance in accuracy and reliability. These measures included (1) step count, (2) walking bout duration, (3) number of total walking bouts, (4) number of long walking bouts, (5) number of short walking bouts, (6) peak 30-minute walking cadence, and (7) peak 30-minute walking pace. To characterize their accuracy, we compared the measure readouts generated from a study device with highly accurate truth labels from an ankle-worn reference device in healthy volunteers. To characterize their reliability, we developed a novel approach to calculate the aggregated time required to reach a reliable readout (time to reliability) for each measure.

## Methods

### Study Participants

The study cohort (pilot program study) included adult volunteer participants, recruited among Verily Life Sciences employees in 2 locations (South San Francisco, CA, and Cambridge, MA), without specific selection criteria. Gender and age information was collected for the accuracy characterization (not for the reliability characterization). This study was determined to be exempt research that did not require institutional review board review.

### Devices

The Verily Study Watch was the study device. This is a wrist-worn smartwatch that records acceleration data via an onboard inertial measurement unit with a 30 Hz 3-axis accelerometer. The study device also has a photoplethysmography sensor and an additional accelerometer and gyroscope with a 100 to 200 Hz sample rate, which this study did not use.

For the accuracy characterization, we used the ankle-worn Modus StepWatch 4, a Food and Drug Administration–listed, 200 Hz 3-axis accelerometer device, as a reference to obtain ground truth labels for step counts (and, subsequently, the other derived walking measures); raw acceleration data from this device were not used for this study. This device has shown the greatest accuracy for step counting relative to other wearable devices compared with human counting in real-world and in-lab settings [24,32].

### Generation of Digital Measurements

We collected continuous, raw accelerometer sensor data from the study smartwatch, computing step counts for every 10-second, nonoverlapping epoch (for additional information about the algorithm associated with the study device to determine step counts, see Multimedia Appendix 1), and collected step count outputs from the reference device (generated by the algorithm associated with the StepWatch) also in 10-second epochs. From the 10-second epoch-based step counts, other measures of walking were derived, applying the same computations to the step counts from both devices (summarized in Table 1). We report the measure readouts as daily aggregates for individual participants (ie, participant-days).

**Table 1:** Summary of walking measure definitions.

Daily walking measure	Definition
Step count	Summed number of steps per day
Number of walking bouts^a^	Summed number of walking bouts per participant-day
Number of short walking bouts	Summed number of walking bouts lasting between ≥30 seconds and <1 minute, per participant-day
Number of long walking bouts	Summed number of walking bouts lasting ≥2 minutes per participant-day
Walking bout duration, mean	Mean duration of daily walking bouts
Walking bout duration, SD	SD of the duration of daily walking bouts
Walking bout duration, 95th percentile	Highest bout duration below the top 5% longest bouts
Peak 30-minute walking cadence^b^	For each participant-day, average cadence for the 180 ten-second epochs (ie, 30 minutes, not necessarily contiguous) with the highest cadence
Peak 30-minute walking pace	For each participant-day, average pace (calculated from the cadence and estimated stride length based on gender and height) for the 180 ten-second epochs (ie, 30 minutes) with the highest cadence (namely, the daily peak 30-minute cadence); measured as meter/second

^a^A walking bout was defined as a series of contiguous 10-second epochs containing ≥6 steps each and lasting for ≥30 seconds (ie, at least 3 epochs). Epochs were considered contiguous if they were not interrupted by >20 seconds (ie, by no more than two 10-second epochs).

^b^Walking cadence was defined as the number of steps per unit of time (in this study, per second—steps/second).

### Analyses

#### Accuracy Characterization

For the characterization of accuracy, the observation period ranged from June 2019 to December 2019. The overall analysis cohort (N=70) was split into 2 equal subcohorts (n=35): training and testing groups (Figure S1 in Multimedia Appendix 1). Participants were required to wear the 2 devices, the smartwatch and the reference device, throughout waking hours for up to 10 days. Step counts were obtained for both the study and the reference devices, for as long as both devices had been worn simultaneously by each participant, and filtered for days with ≥8 hours of wear time and >100 steps. Each subsequent measure was derived based on step counts from each device (Table 1) and compared for agreement. Agreement was examined using the following metrics: Fisher intraclass correlation coefficient (ICC) as the main metric, Pearson *r* coefficient, mean error, and mean absolute error. For each metric, we calculated 95% CIs by bootstrapping with 1000 resampling iterations to account for multiple days (generally 5) from a given participant. Additionally, to further characterize the degree of agreement and bias of each measure, we examined measurements and distributions between devices and Bland-Altman plots with 95% limits of agreement.

#### Reliability Characterization

For time-to-reliability characterization, the observation period was 20 months (April 13, 2018, to December 31, 2019). This analysis was designed to determine the duration of time (from 1 to 30 days) over which each measure needs to be aggregated (the different lengths of time tested were termed “aggregation scopes”) to yield stable values, indicating that it reliably captures an individual’s underlying behavioral state. Data were considered analyzable for this objective when participants had worn the device for at least double the duration of a given aggregation scope (in order to have data for 2 nonoverlapping time windows), starting from a minimum of 2 days (for the shortest aggregation scope of 1 day) to a minimum of 60 days (for the longest aggregation scope of 30 days); in addition, at least 50% of the days in each time window had to have ≥12 hours of daily wear. The number of participants meeting these criteria varied according to the span of the aggregation scopes (N=234 for the 1-day aggregation scope [ie, the smallest aggregation scope had the largest cohort]; n=81 for the 30-day aggregation scope [smallest cohort for the largest aggregation scope]; Figure S1B in Multimedia Appendix 1).

In this analysis, we included the same set of measures as for the accuracy characterization, except the 30-minute peak walking pace, because the measure is derived directly from 30-minute peak walking cadence (Table 1); therefore, the results of this analysis were expected to be identical between these 2 measures. We calculated Fisher ICCs between adjacent, nonoverlapping windows of time for each aggregation scope (1-30 days). We computed a rolling mean for each daily aggregated measure over the set number of days for each aggregation scope and then computed the ICC between adjacent windows. We repeated this computation by shifting the start date of each window by 1 day and repeated the computation testing aggregation scopes between 1 and 30 days.

## Results

### Accuracy Characterization

A total of 162 participant-days worth of data were collected from the 35 participants in the test cohort, with each participant contributing 1 to 10 days (median 5 days). The mean daily step count, daily ambulatory time, and wear time per participant-day were 10,075.88 (SD 4321.07) steps, 1.86 (SD 0.78) hours, and 13.73 (SD 3.00) hours, respectively (Figure S2 in Multimedia Appendix 1).

**Figure 1:**
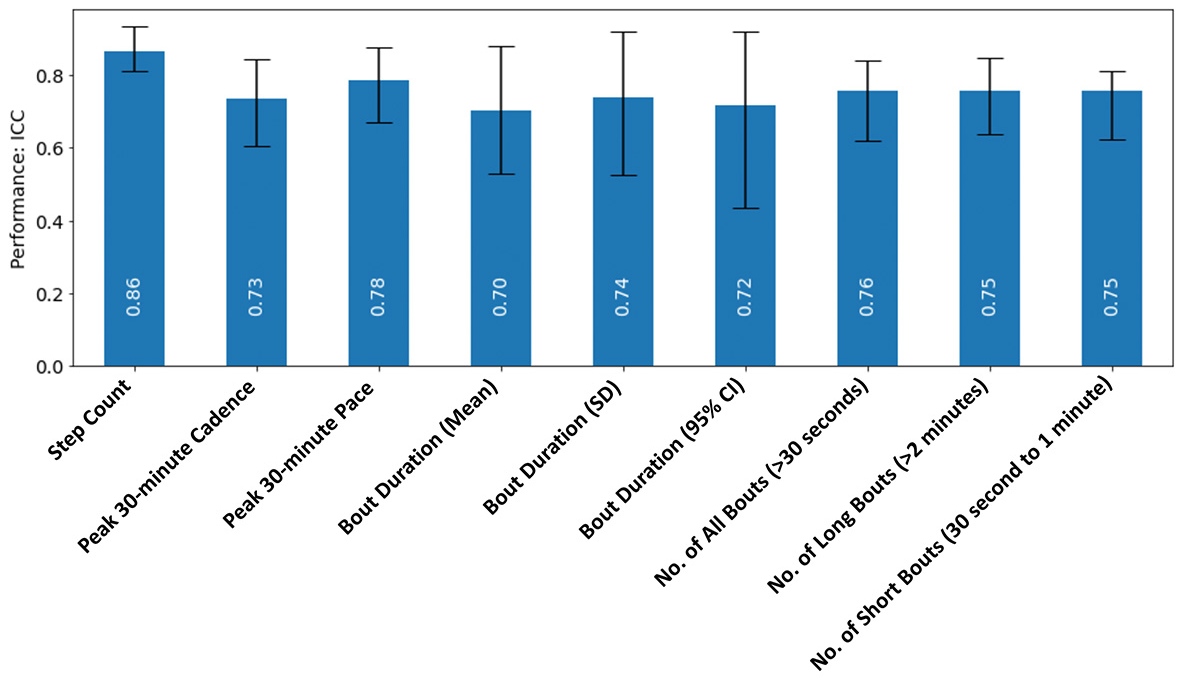
Accuracy characterization: ICC results (and 95% CIs) obtained from the comparison of the digital measurements generated from the study device against those from the reference device. ICC: intraclass correlation coefficient.

**Figure 2:**
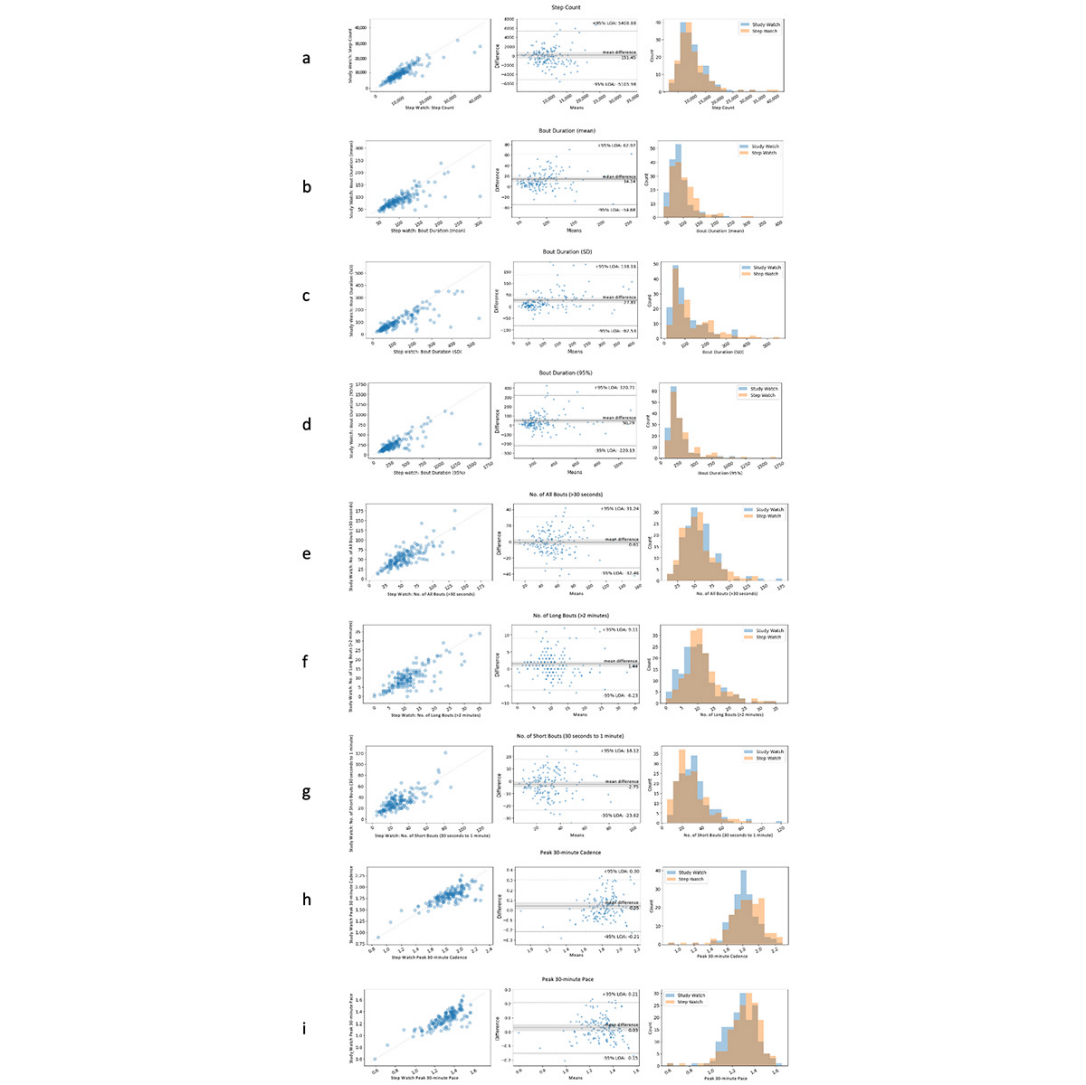
Accuracy characterization: detailed results of the comparisons of the digital measures generated from the study device against those from the reference device. Left column: plots of study device readouts (y-axis) versus reference device readouts (x-axis). Middle column: modified Bland-Altman plot showing the difference in mean values between devices (y-axis) versus mean values from the reference device (x-axis). Right column: readout value distributions for both devices in the testing subcohort. (A) Daily step count. (B) Daily walking bout duration, mean. (C) Daily walking bout duration, SD. (D) Daily walking bout duration, 95th percentile. (E) Number of daily walking bouts. (F) Number of daily long walking bouts. (G) Number of daily short walking bouts. (H) Daily peak 30-minute walking cadence. (I) Daily peak 30-minute walking pace. LoA: limits of agreement. See Multimedia Appendix 2 for higher resolution image.

For each measure of interest (see the Methods section), the comparison of the values generated from the study device against the reference device showed ICC values ranging between 0.701 and 0.865 (Table 2, Figure 1); the measure “mean duration of daily bouts” produced the lowest ICC value (0.701), and “daily step count” had the highest (0.865). Pearson *r* values were all greater than 0.75, and all values were statistically significant (*P*<.001; Table 2). The Bland-Altman analysis (Figure 2, middle) revealed that measure differences between the study and reference devices were not dependent on the measure value without significant bias. Scatter plots (Figure 2, left) and distribution (Figure 2, right) of measures between study and reference devices showed overlap for all 9 measures of walking.

**Table 2:** Summary of results from the characterization of accuracy of the measures generated from the study device compared with those collected from the reference device (N=35).

Accuracy metric	ICC^a^ (95% CI)	Pearson *r* (95% CI)	ME^b^ (95% CI)	MAE^c^ (95% CI)
Daily step count	0.865 (0.809 to 0.933)	0.881 (0.832 to 0.941)	151.450 (–486.869 to 726.201)	1643.145 (1196.832 to 1996.216)
Mean duration of daily bouts	0.701 (0.529 to 0.876)	0.784 (0.657 to 0.922)	14.143 (9.657 to 17.607)	17.141 (12.985 to 20.371)
Daily bout duration, SD	0.738 (0.525 to 0.918)	0.813 (0.659 to 0.948)	27.813 (17.180 to 36.224)	32.585 (22.038 to 41.081)
95th percentile of daily bout duration	0.715 (0.433 to 0.918)	0.763 (0.550 to 0.932)	50.293 (28.720 to 67.285)	69.620 (49.051 to 85.290)
Number of daily bouts	0.756 (0.620 to 0.838)	0.757 (0.632 to 0.848)	–0.611 (–3.740 to 2.840)	11.846 (9.743 to 13.698)
Number of long daily bouts	0.755 (0.638 to 0.845)	0.781 (0.671 to 0.861)	1.438 (0.780 to 2.103)	2.858 (2.426 to 3.232)
Number of short daily bouts	0.754 (0.621 to 0.811)	0.768 (0.648 to 0.830)	–2.747 (–4.553 to –0.736)	8.401 (7.172 to 9.542)
Daily peak 30-minute cadence	0.734 (0.603 to 0.841)	0.773 (0.662 to 0.862)	0.045 (0.013 to 0.076)	0.100 (0.080 to 0.118)
Daily peak 30-minute pace	0.784 (0.668 to 0.873)	0.802 (0.710 to 0.877)	0.030 (0.007 to 0.051)	0.070 (0.057 to 0.082)

^a^ICC: intraclass correlation coefficient.

^b^ME: mean error.

^c^MAE: mean absolute error.

### Reliability Characterization

In the cohort of eligible participants who yielded analyzable data (see the Methods section, N=234), individual participant data were collected for up to 596 (median 119) days for a total of 15,120 participant-days (see Figure S1B in Multimedia Appendix 1). The mean daily step count, daily ambulatory time, and daily wear time per participant-day were 9701.06 (SD 4321.88) steps, 77.42 (SD 39.77) minutes, and 17.36 (SD 4.04) hours, respectively.

**Figure 3:**
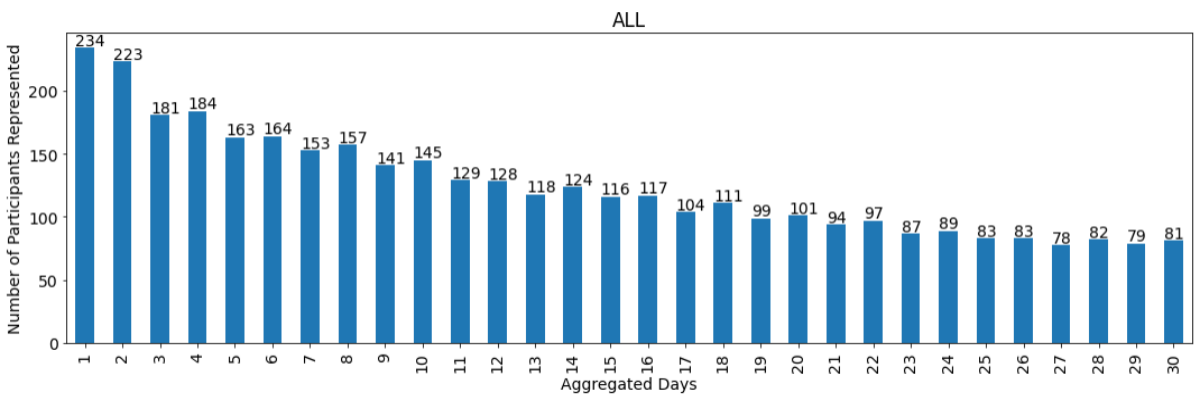
Time-to-reliability characterization: size of the participant subcohorts with analyzable data across the aggregation scopes tested.

We defined aggregation scopes of increasing duration from 1 day up to 30 days. For each of these scopes, the participant subcohorts that generated data deemed analyzable were of variable size (generally decreasing as the aggregation scope grew, Figure 3).

**Figure 4:**
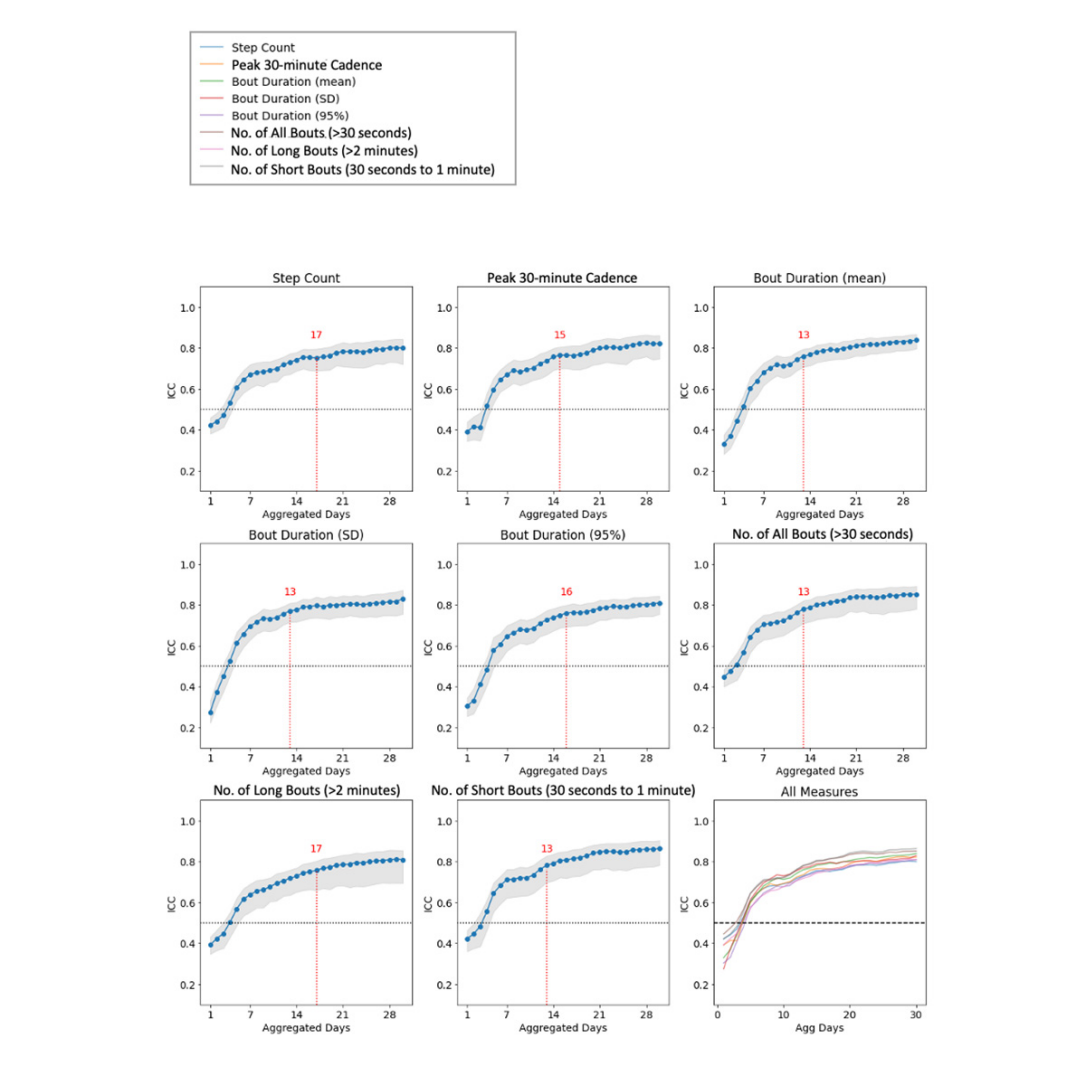
Time-to-reliability characterization: ICCs between adjacent readout windows according to aggregation scope duration, for the digital measures of interest. The line represents the ICC value plot, gray shading represents 95% CIs, and red annotations indicate the aggregation scope first exceeding an ICC value of 0.75. ICC: intraclass correlation coefficient. See Multimedia Appendix 3 for higher resolution image.

Across all the measures of interest in this analysis, the stability of the measure (estimated using ICC between adjacent time windows for readout) increased with longer aggregation scopes. The metrics “number of daily bouts,” “bout duration, SD,” and “number of short bouts” reached an ICC ≥0.75 at the earliest aggregation scope (12 days). Ultimately, all digital measures achieved an ICC ≥0.75 by 16 days, which we defined as the potential time-to-reliability benchmark in the context of this study (Figure 4). ICCs reached a plateau at values ranging between 0.78 and 0.84, depending on the measure.

## Discussion

This report expands upon prior research [30,31], presenting a comprehensive application of an algorithm that captures step count and other aspects of mobility, such as walking cadence and bouts. We characterized the accuracy and reliability of this comprehensive set of digital walking measures from users wearing a wrist-worn device in real-world environments. We showed that these measures of walking reached reliable readings at around 16 days of wear time, and their levels of agreement with the reference device, measured by ICC, ranged between 0.7 and 0.9, a performance that supports their deployment in clinical trial settings with confidence.

Mobility and walking behaviors represent meaningful aspects of health, known to be associated with quality of life in general and clinical prognosis in specific settings [33-35]. Therefore, improved methods to measure mobility and walking behaviors have the potential to improve clinical care and clinical trial efficiency. One of the goals of our research is to build accurate tools to objectively quantify the aspects of walking behavior and extract clinically meaningful information in discrete populations of interest. In prior work, we have developed algorithms whose outputs (measures for step counts and ambulatory time) demonstrated sensitivity to treatment effects in patients with Lewy body dementia [31]. We have also characterized the accuracy of an iteration of that algorithm in a cohort of diverse individuals in the real world [30].

To our knowledge, this report is the first to characterize the amount of data required to ensure that a digital measure is reliable in a real-world setting. We developed a novel analytic approach to characterize time to reliability, that is, the time needed for a measure to reach a degree of stability. Time to reliability is an important consideration to inform the design of clinical studies tracking real-world data, as it relates to specific metrics of interest. In this study, the time to reliability overall for all measures was ≤16 days (ICC ≥0.75 between nonadjacent readouts for all measures at day 16, Figure 4). This study included healthy individuals; in a clinical context, we anticipate that the stability of any given measure over time will be dependent on the type and severity of the disease of interest. It is reasonable to speculate that a mostly healthy cohort may demonstrate more variability and a larger distribution of walking behaviors than a cohort with disease burden, and this necessitates further research.

Most importantly, the algorithms developed to quantify daily step count and measures related to walking cadence and bouts were found to be accurate (agreement between the readouts from the study device and a highly accurate reference device ranged between ICCs of 0.7 and 0.9 for all measures, Table 2). Our study approach captured the measures of walking behavior in a real-world setting, over multiple days, to closely resemble actual use cases.

Considering the exponential growth of research on wearable sensors and related devices in recent years, it is important to place the capabilities described in this report in that context. In this work, we incorporated several key innovative approaches to address shortcomings present in comparable studies evaluating interdevice agreement. Prior studies have used colocated investigational and reference sensors (eg, 2 wrist-worn devices). But because of the known potential errors associated with body placement when capturing walking-related data [36-39], colocation could be vulnerable to bias toward overestimating performance. Our approach sought to mitigate that by using a highly accurate but pragmatic and ankle-worn source for ground truth labels. Further, most studies have narrowly focused on step counts [10-16] for short time periods in controlled laboratory environments (eg, only a single day in real-world settings), or when investigating walking bout and cadence or pace measures, they had a limited scope, with small samples of less than 40 participants [40-42] and short tests (sessions lasting 1 hour or less) performed in clinic. Our study addresses these existing evidence gaps, presenting a set of digital walking measures that are comprehensive beyond step counts and characterizing their analytic performance (accuracy and reliability) extensively, with data accrued throughout multiday periods and in the course of daily living activities. Moreover, given the research heterogeneity (comparisons of different devices, different ground truth sources, and with different analytic approaches), any direct comparison of study results side by side has to be done with caution, which highlights the need for standardization noted in professional statements in this field [6,9,43,44].

This study had limitations in regard to the participant population and the performance quality thresholds. First, our cohort was limited in size and consisted of generally healthy participants. Future studies may be needed to characterize the generalizability of the performance of these measures in populations with particular kinetic hallmarks (eg, neurological conditions, stroke, and trauma) or with mobility capacity issues (eg, cardiovascular or respiratory conditions). Our approach to determine time to reliability can be applied across studies in any therapeutic area and can guide study design requirements for wear time compliance. One aspect that will require attention is the optimization of actual compliance with hypothetical protocol specifications about wear time because this is a device intended for daily life use (for instance, our reliability analysis filtered participant-day data based on a threshold of 12 hours of daily wear time for 50% of the days over an evaluation period, but we did so retrospectively). Second, although we report on performance parameters, the definition of an acceptable performance (accuracy and reliability) quality threshold remains undefined in the field. We did not prespecify performance categories in this study, but, for instance, prior accuracy studies have categorized agreement ICC values 0.7-0.9 as moderate to good [40,45], and reliability studies for patient-reported outcomes have considered test-retest ICC >0.5 as acceptable [46]. Importantly, what constitutes a clinically meaningful change for each of the measures of walking will likely depend on the therapeutic area under consideration. Further research is also needed to address which of these measures can provide clinically relevant insights in a given population. This set of digital walking measures has the potential to convey comprehensive information beyond flat quantification (via step counts), about aspects such as maximal walking capacity, endurance, or activity patterns during daily living, which may have different relevance or sensitivity to detect status changes depending on the health setting (for instance, cardiopulmonary conditions, oncology, or neurology). Furthermore, the optimization of the clinical utility of these measures may require their aggregation into composite metrics. The potential complexity of this future research brings to the forefront the importance of establishing first a thorough understanding of their individual analytical performance, which this study does. We believe that the accuracy and reliability results detailed here are the first step to support the use of digital measures of walking as feasible and reliable end points in clinical studies.

In conclusion, we have developed algorithms that accurately quantify daily step counts and measures of walking cadence and bouts from users wearing a wrist-worn device in a real-world setting. Further, we have also developed a novel method for characterizing the time required for a digital measure to stabilize (time to reliability). Given the growing use of wearable sensors to measure aspects of health, these findings may guide practical implementation of these digital measures of walking behavior into clinical studies.

## Appendix

Multimedia Appendix 1 Additional methods and results.

Multimedia Appendix 2 High resolution version of Figure 2.

Multimedia Appendix 3 High resolution version of Figure 4.

## Abbreviations

ICC: intraclass correlation coefficient

## Data Availability

Data from this study are not available due to the nature of this program. Participants did not consent for their data to be shared publicly.
