# Supplementary material for: Accuracy and Reliability of a Suite of Digital Measures of Walking Generated Using a Wrist-Worn Sensor in Healthy Individuals: Performance Characterization Study
Source: JMIR Hum Factors. 2023 Aug 3;10:e48270. doi: 10.2196/48270 (PMC10436116; doi:10.2196/48270)
Supplement: Multimedia Appendix 1 [file humanfactors_v10i1e48270_app1.docx]

## **Multimedia Appendix 1**

**Step Count Algorithm for the Verily Study Watch**

The underlying algorithm processes device user data in 10-second epochs. We trained a neural network on StepWatch generated ground-truth labels (n=35, same dataset as Pilot Program in Figure S1). The following 14 features were extracted from the study device acceleration data, in 10-second epochs: 3 features related to deviations of the signal, 5 features derived from the power spectral density (PSD) energy in frequency bands typically associated with user’s ambulation (eg, walking or running), 2 features that are signal percentiles (eg, 95th percentile), and 4 features that are differences between signal percentiles (eg, interquartile range). Detailed validation results of the ambulatory status classification algorithm were published previously [1].

**Figure S1.** Participant flow into study cohorts/subcohorts

(A) Characterization of accuracy, participant flow

**PILOT COHORT**

**Overall**

**N=75**

**Evaluable**

**N=70**

**n=5**

Wear time insufficient or asynchronous

**Pilot Held-out Test**

**n=35**

**Pilot Training**

**n=35**

(B) Time-to-reliability characterization, participant flow

**PILOT COHORT**

**Overall**

**N=271**

**Evaluable**

**N=234**

- Worn the device required minimum numbers of days
- Worn the device at least 12 h 50% of the days in the time windows for each aggregation scope

**30 days**

**n=81**

**1 Day**

**n=234**

*Allocation into aggregation scope analyses:*

**…….**

**Table S1.** Characteristics of the participants in the cohort for the accuracy characterization

|  |  | Accuracy Characterization Cohort (N=75) |
| --- | --- | --- |
| Sex, n (%) | Female | 25 (35.7) |
|  | Male | 45 (64.3) |
| Age, yrs | Median | 30 |
|  | Mean (SD) | 33.2 (8.5) |
|  | Range | 23 - 63 |

**Figure S2.** Characterization of accuracy: Overall features (in terms of distribution of walking activities) of the data collected from the testing subcohort.

(A) For each individual participant, total number of device-wearing days, and total number of ambulatory hours.


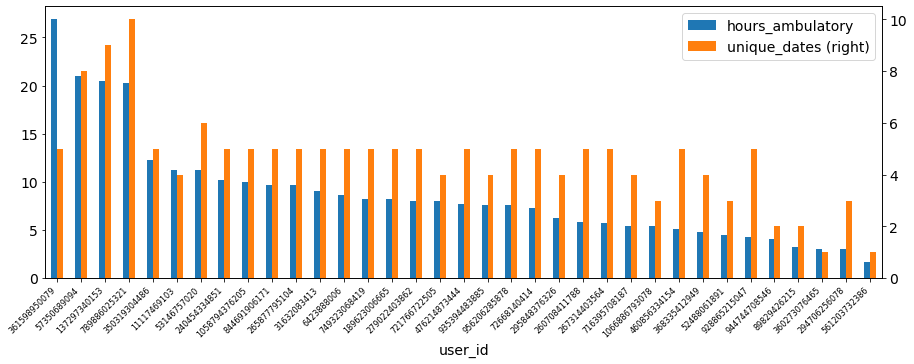


(B) Distribution of daily ambulatory times (top) and daily step counts (bottom), per participant-day


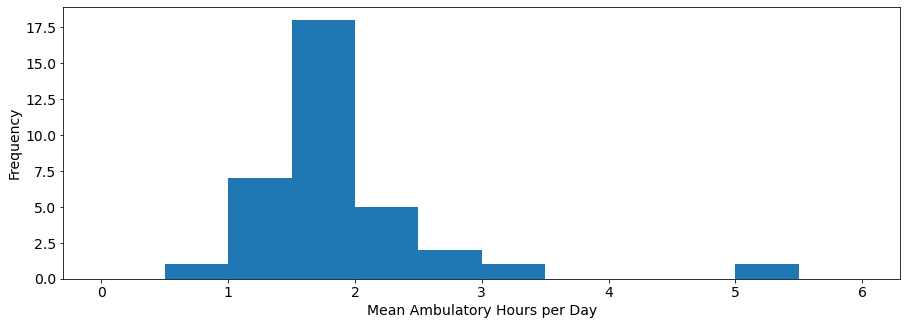


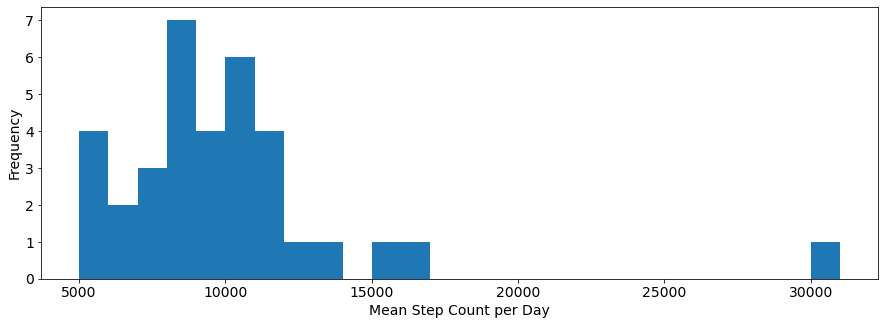


**Reference**

1. Popham S, Burq M, Rainaldi EE, Shin S, Dunn J, Kapur R. An algorithm to classify real-world ambulatory status from a wearable device using multimodal and demographically diverse data: Validation study. JMIR Biomed Eng. 2023;8(1):e43726. DOI: 10.2196/43726
