# Supplementary figures and images for: Accuracy and Reliability of a Suite of Digital Measures of Walking Generated Using a Wrist-Worn Sensor in Healthy Individuals: Performance Characterization Study
Source: JMIR Hum Factors. 2023 Aug 3;10:e48270. doi: 10.2196/48270 (PMC10436116; doi:10.2196/48270)

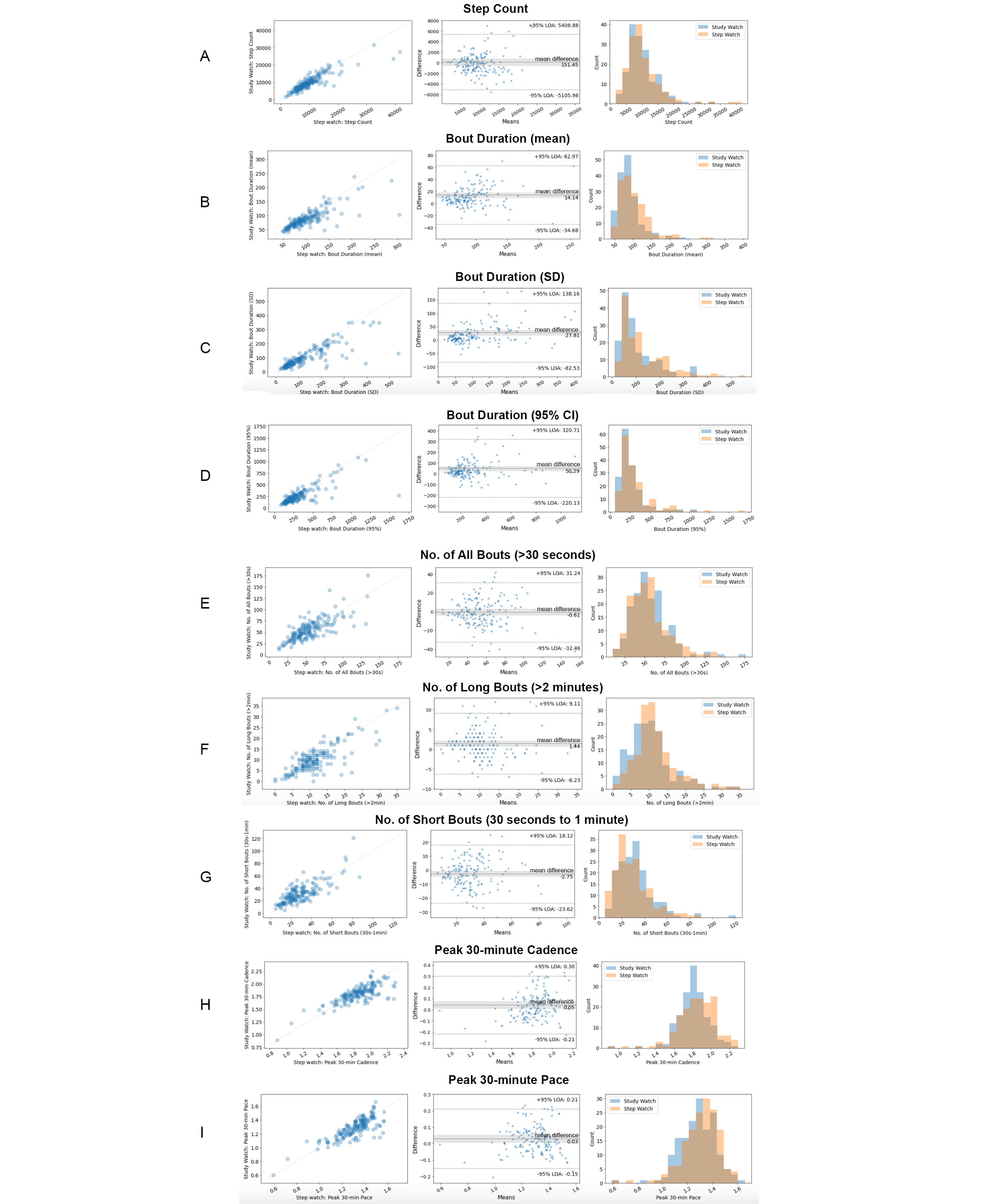

Supplement: Multimedia Appendix 2 [file humanfactors_v10i1e48270_app2.png]

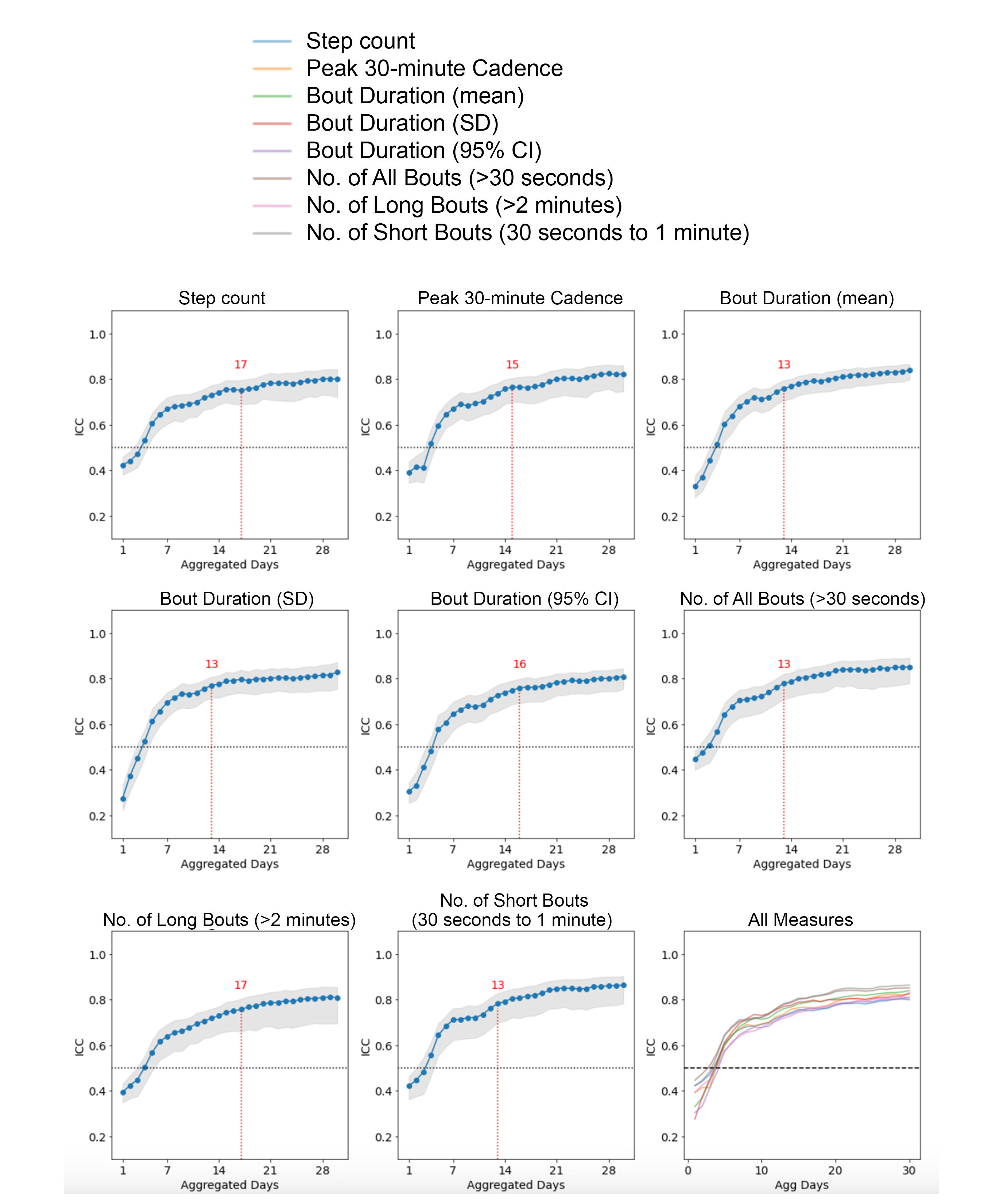

Supplement: Multimedia Appendix 3 [file humanfactors_v10i1e48270_app3.png]
